# Health care quality in a new Emergency Department based on the Danish Stroke register data

**DOI:** 10.1186/1757-7241-21-S2-A27

**Published:** 2013-09-09

**Authors:** Maria Søe Mattsson, Michael Oettinger, Hanne Jørsboe

**Affiliations:** 1 Emergency Department, Nykøbing F. hospital, Denmark

## Background

One of the intentions to develop the concept of an ED in Denmark is to increase health care quality in the treatment of acute patients. However, it is a massive reorganization including other workflows and competency profiles. At present, there are not established any general quality indicators for the acute treatment, but hospitals have reported to the Danish Stroke Register (DAP) for selected diseases. We have chosen “Stroke” as case to evaluate quality during a 3 years period under implementation of the ED concept, since these patients are among the 20 most common illnesses in our department.

## Methods

The study is quasi-experimental. All patients with Stroke from the ED at Nykøbing F. Hospital, which reported to DAP since 2007, are included in the study. Period 2007/2008 works as a historical control. The ED started at April 2009. In the study, indicators are chosen to describe early interventions in the patient pathway and compare them with nationwide data. Data is processed in STATA and Chi2-test is used to analyze whether there has been a change over time.

## Results

1715 patients entered the study. Gender and age are comparable. Analysis shows that concerning the indicator CT/MR scan within 24 hours, there has been a improvement both in the ED (50% in 2007/2008, 55% in 2009, 65% 2010, 76% in 2011, 83% in 2012, p <0,005) and nationally (67% in 2007/2008 to 85% in 2011). Similarly, the indicator concerning treatment with antiplatelet therapy, improvement are made in both the ED (50% in 2007/2008, 55% in 2009, 65% 2010, 76% in 2011, 83% in 2012, p <0,005) and nationally (87% in 2007/2008 to 93% in 2011). Relative to mortality within 30 days, there has been an improvement in the ED (13% in 2007/2008, 9% in 2009, 11% 2010, 8% in 2011, 5% in 2012, p <0,005) but not nationally (10% in 2007/2008 to 11% in 2011).

## Conclusion

During establishment of an ED, the treatment of stroke has improved reflecting earlier diagnosis and treatment. The results are comparable to nationwide results from other organisational setup. It is recommended, that indicators for monitoring acute treatment in Denmark is developed.

